# Deep brain stimulation for treatment resistant obsessive compulsive disorder; an observational study with ten patients under real-life conditions

**DOI:** 10.3389/fpsyt.2023.1242566

**Published:** 2023-09-13

**Authors:** Mohamed A. Abdelnaim, Verena Lang-Hambauer, Tobias Hebel, Stefan Schoisswohl, Martin Schecklmann, Daniel Deuter, Juergen Schlaier, Berthold Langguth

**Affiliations:** ^1^Department of Psychiatry and Psychotherapy, University Regensburg, Regensburg, Germany; ^2^Center for Deep Brain Stimulation, University Regensburg, Regensburg, Germany; ^3^Department of Psychology, University of the Bundeswehr Munich, Neubiberg, Germany; ^4^Department of Neurosurgery, University Regensburg, Regensburg, Germany

**Keywords:** OCD, DBS, BNST, invasive brain stimulation, treatment-resistant obsessive-compulsive disorder

## Abstract

**Introduction:**

Obsessive-compulsive disorder (OCD) affects 2–3% of the global population, causing distress in many functioning levels. Standard treatments only lead to a partial recovery, and about 10% of the patients remain treatment-resistant. Deep brain stimulation offers a treatment option for severe, therapy-refractory OCD, with a reported response of about 60%. We report a comprehensive clinical, demographic, and treatment data for patients who were treated with DBS in our institution.

**Methods:**

We offered DBS to patients with severe chronic treatment resistant OCD. Severity was defined as marked impairment in functioning and treatment resistance was defined as non-response to adequate trials of medications and psychotherapy. Between 2020 and 2022, 11 patients were implanted bilaterally in the bed nucleus of stria terminalis (BNST). Patients were evaluated with YBOCS, MADRS, GAF, CGI, and WHOQOL-BREF. We performed the ratings at baseline (before surgery), after implantation before the start of the stimulation, after reaching satisfactory stimulation parameters, and at follow-up visits 3, 6, 9, and 12 months after optimized stimulation.

**Results:**

One patient has retracted his consent to publish the results of his treatment, thus we are reporting the results of 10 patients (5 males, 5 females, mean age: 37 years). Out of our 10 patients, 6 have shown a clear response indicated by a YBOCS-reduction between 42 and 100 percent at last follow-up. One further patient experienced a subjectively dramatic effect on OCD symptoms, but opted afterwards to stop the stimulation. The other 3 patients showed a slight, non-significant improvement of YBOCS between 8.8 and 21.9%. The overall mean YBOCS decreased from 28.3 at baseline to 13.3 (53% reduction) at the last follow-up. The improvement of the OCD symptoms was also accompanied by an improvement of depressive symptoms, global functioning, and quality of life.

**Conclusion:**

Our results suggest that BNST-DBS can be effective for treatment-resistant OCD patients, as indicated by a reduction in symptoms and an overall improvement in functioning. Despite the need for additional research to define the patients’ selection criteria, the most appropriate anatomical target, and the most effective stimulation parameters, improved patient access for this therapy should be established.

## 1. Introduction

Obsessive-compulsive disorder (OCD) occurs in the form of repetitive intrusive thoughts and/or actions, which are typically experienced as uncomfortable and often considered as nonsensical. Patients’ attempts to resist these thoughts or actions are mostly unsuccessful. For OCD, the lifetime prevalence is about 2–3% worldwide (1).

OCD causes significant distress, is time-consuming, and significantly interferes with the person’s normal daily routine, work (or school) functioning, or usual activities and relationships (2).

OCD is highly co-morbid with other mental illnesses (3). It has been reported that 90% of those who meet the criteria for OCD also meet the criteria of at least one other mental disorder over the course of their life (1). Frequent comorbidities of OCD include mood disorders and anxiety disorders (4).

The standard treatment for OCD consists of a combination of psychotherapy and pharmacotherapy, but even with these treatments, about 40–60% of patients experience only partial recovery, and about 10% of OCD patients remain treatment resistant, which leads to significant functional limitations (5–7).

One treatment option for severe, therapy-refractory obsessive-compulsive disorders represents deep brain stimulation (DBS). In DBS, electrodes are stereotactically implanted unilaterally or bilaterally in specific brain regions, which can then be stimulated via a battery located under the skin of the upper chest. This method has already proven remarkable benefits for people with a variety of neurologic conditions, and many researchers have investigated the potential benefit of DBS of selected brain regions for other disorders such as pain, depression, and obsessive compulsive disorder (8). Bilateral DBS of the anterior limb of the internal capsule (ALIC) has shown remarkable effects in patients with treatment resistant OCD (9, 10) and based on these data, the U.S. Food and Drug Administration (FDA) approved in 2009 DBS for treatment-refractory OCD as a humanitarian device exemption (HDE H050003) (11).

The exact mechanism of action of DBS in OCD is not fully understood. Since OCD might be explained through abnormal activity in corticostriatal-thalamocortical (CSTC) circuitry (12), it is hypothesized that DBS may disrupt the CSTC circuit, thereby restoring normal connectivity within and between circuits (13). Also, it has been postulated that DBS induces release of neurotransmitters, such as GABA and glutamate (14, 15). The role of the later in pathogenesis, clinical manifestations and treatment response of obsessive-compulsive disorder has been supported by evidence from animal studies, neurophysiological studies, genetic, neuroimaging studies (16). Accordingly glutamate-modulating medications have been investigated as a treatment option for OCD (17).

The effectiveness of DBS in OCD has been examined in several studies, targeting different brain regions. Mainly, the anterior limb of internal capsule (ALIC) (18–20), the bed nucleus striae terminalis (BNST) (21), ventral capsule/ventral striatum (VC/VS) (22–24), the nucleus accumbens (NA) (25–27), and the nucleus subthalamicus (STN) (28) have been targeted. The efficacy of DBS for OCD patients has been evidenced by many further studies (29, 30), summarized in various systematic reviews (31–34). Meta-analyses reported statistically significant effects of DBS in treatment-resistant OCD patients (35, 36). Alonso et al. estimated a mean reduction of the Yale–Brown Obsessive Compulsive Scale (Y-BOCS) score of 45.1% and a global percentage of responders of 60.0% (37). Moreover, long-term data demonstrated sustained improvement for the responders (38–46). Despite these impressive results, only very few potential candidates receive DBS for OCD (47). According to recent systematic reviews, only data from about 350 patients is reported in the world literature (48). Moreover, the investigated stimulation targets vary considerably across studies.

In this context, a systematic collection of clinical and demographic data of further patients would be highly desirable, to gain more knowledge about efficacy and safety, the importance of the stimulation target, successful stimulation settings, the relevance of medication as well as the significance of predictors for response and effects of DBS treatment on patients’ co-morbidities. Thus, in order to enlarge the knowledge base of DBS for OCD we report comprehensive clinical, demographic, and treatment data from 10 consecutive patients from our institution.

## 2. Patients and methods

All patients presented have provided written informed consent to this observational study, which was approved by the ethic committee of the University of Regensburg (ethic vote: 21-2707-104). Observational means that DBS was not the issue of the study but the systematic evaluation of the change in patients’ pathology after treatment. All patients, who underwent DBS for their OCD between January 2020 and December 2022 at the multidisciplinary center of deep brain stimulation at the University of Regensburg, Germany, were included in the study.

### 2.1. Patients’ selection

Potential candidates for DBS were screened for their eligibility first at the outpatient clinic of the department of psychiatry and psychotherapy and then at the outpatient clinic of the department of neurosurgery.

#### 2.1.1. Objectives

The objectives of the screening assessment were as follows;

a) confirmation of the OCD diagnosis through obtaining a comprehensive patient history, and by checking all available health records.
b) getting a chronological summary of previous treatment trials, including pharmacotherapies, psychotherapies, or other interventions.
c) collecting information on the patient’s psychosocial history and overall functioning.
d) to review the patient’s eligibility for DBS in accordance with certain in-and exclusion criteria as listed below.
e) In addition to a comprehensive assessment of interested candidates by two different psychiatrists, patients’ treating psychiatrists were contacted for complementary information.

#### 2.1.2. Inclusion and exclusion criteria

1. Age (18 or older).
2. Chronicity, defined as at least five years of OCD without remission.
3. Treatment resistance, defined as fulfilling the following criteria;

- Non-response to adequ*ate trials with a maximum tolerated dose of at least two different serotonine reuptake inhibitors (SSRI) and one trial with clomipramine or* augmentation with an antipsychotic (risperidone or aripiprazole).
- Non-response to CBT for at least one year (>50 sessions), including exposure therapy.
- Non-response to an adequate multi-professional treatment procedure (e.g., inpatient clinic with different therapy modalities).

4. Regarding severity, we did not rely only on YBOCS scores and therefore did not define a cut-off; we rather considered overall impairment in social, occupational functioning and patient’s normal routine.
5. Exclusion of other relevant (dominant) psychiatric disorder; esp. psychotic disorder, substance abuse/dependency disorder, or personality disorder.
6. Exclusion of current clinically significant neurological disorder or medical illness.
7. Exclusion of clinically significant abnormality or any medical contraindication to DBS surgery.
8. Exclusion of acute suicidality.

### 2.2. Surgery

Patients, who fulfilled the criteria for DBS in the psychiatric assessment, were referred to the neurosurgery department, where their eligibility from a surgical point of view was evaluated. Patients received a detailed information about the operative process. All patients gave informed written consent to the surgical procedure, and the operation was only performed after a sufficient consideration period of at least 60 days.

Two days prior to the operation, preoperative MR imaging was performed at a 3 T SIEMENS Magnetom Skyra scanner with patients under general anesthesia during the whole imaging to avoid movement artefacts in preparation of DBS surgery. Sagittal T1 and axial and sagittal T2 images parallel to the intercommisural plane were acquired for the planning of the trajectory as well as T1 + double dose Gadolinium images to visualize crucial blood vessels to avoid bleeding when inserting stylets and DBS electrodes. On the date of surgery, a preoperative CT-scan with a stereotactic frame mounted on the patient’s head (CRW, Integra Radionics, Burlington, United States) obtained from a SIEMENS Somatom Definition Flash scanner served as reference for surgery planning. Trajectories avoiding relevant blood vessels, sulci and crucial neurological structures were defined using iPlanNet 3.0 (BRAINLAB, Munich, Germany) with targets in the bed nucleus striae terminalis (BNST). The stereotactic implantation of the electrodes (3,391, 3,387, or B3301533; Medtronic plc, Dublin, Ireland) and the implantation of the internal pulse generator (IPG) (ActivaRC or PerceptPC; Medtronic plc, Dublin, Ireland) was performed in one setting with the patient under general anesthesia. Postoperatively, electrode position was controlled by CT scans with 1 mm slice thickness, which were fused to the MR imaging.

### 2.3. Stimulation

Stimulation was normally initiated 6–8 weeks following surgery and was titrated by a psychiatrist with experience in DBS. First, bilateral stimulation of each of the four contacts was tested for tolerability and efficacy. Then, at the best contacts voltage was stepwise increased to achieve the best therapeutic efficacy. After reaching optimal voltage, further optimization of the other stimulation parameters (frequency, pulse width) was performed. If the target effectiveness was not attained, the same procedure was performed with the second-best contact.

### 2.4. Assessment

#### 2.4.1. Assessment tools

Y-BOCS was used to evaluate the existence and severity of OCD symptoms (49), which measures the severity of symptoms of OCD based on scores of obsessions and compulsions. The Y-BOCS checklist was used for assessing present and past OCD symptoms (50). For assessing the degree of functioning, severity and improvement, the Global Assessment of Functioning (GAF) (51) as well as the Clinical Global Impressions Scale (CGI) (52) were used. Quality of life was assessed with WHO-quality of life Questionnaire (the four dimensional WHOQOL-BREF “physical, psychological, social, and environment”) (53). For some patients, the Montgomery-Asberg Depression Rating Scale (MADRS) was performed to measure the severity of depressive symptoms (54).

#### 2.4.2. Assessment time-points

We performed the ratings at baseline, after surgery but before start of stimulation (before stimulation), then after reaching a satisfactory stimulation parameters (optimized stimulation) and at follow-up visits 3, 6, 9, and 12 months after reaching the optimized stimulation parameters.

#### 2.4.3. Titration (optimization) visits

During the optimization period, i.e., search for optimal stimulation parameters, patients were regularly asked about their subjective feeling, improvement/worsening of OCD-symptoms, as well as side effects.

### 2.5. Analysis of results

All statistical analyses were performed using the software R (R version 4.2.3; R Foundation for Statistical Computing, Vienna, Austria). To evaluate symptom changes over study visits (baseline, before stimulation, optimized stimulation, 3 months follow-up, 6 months follow-up, 9 months follow-up, and 12 months follow-up), linear mixed effect models were applied for each assessment inventory. Thereby, the study visit was always treated as a fixed effect and the individual patient as a random effect. The effect of study visit was assessed via the expected mean square approach and in case of a significant effect, *post hoc* Tukey contrasts were used to analyze potential score differences between study visits. *Post hoc* results were adjusted for multiple comparisons using the Tukey method. The level for statistical significance was set at 5%.

## 3. Results

Eleven patients have fulfilled our criteria, were identified as eligible candidates and were implanted between January 1st, 2020 and December 31st 2022. One patient has retracted his consent to publish the results of his treatment, thus his data are not included in the analysis. We are reporting the results of 10 patients (5 males, 5 females, age between 20 and 63 years “mean: 37 years”). Since the patients were implanted at different times and some of them missed visits, the number of patients varies for every visit. The demographic data are summarized in Table 1.

**Table 1:** Demographic data of patients.

Patient Number	Age at presentation	Sex	Family and employment status	Age of onset of OCD	Comorbidities	Main OCD-symptoms and YBOCS (obsessions, compulsions)	Previous therapies
1	35	Female	In relationship, disability pension	14	Depression, GAD	Intrusive thoughts, repeated questioning YBOCS at presentation: 18 (18,0).	SSRIs: sertraline, escitalopram. SNRI: venlafaxine. Milnacipran. clompiramine (not tolerated), amitriptyline, augmentation of antidepressants with risperidone, quetiapine, haloperidol. CBT sessions for many years, also psychoanalysis. 3 inpatient treatment in different psychiatric clinics.
2	25,	Male	Single, unemployed	6	Asperger, ADHD	Obsessions about controlling, perfectionism, repeated checking, fear of contamination. YBOCS at presentation: 37 (20,17)	Many medications in different combinations. Over 3 years of psychotherapy (CBT). 3 inpatient treatments.
3	29	Female	Single, unemployed	23	Depression	Fear of losing items or mistakes, repeated controlling and checking. YBOCS at presentation: 31 (18,13)	3 SSRIs trials all with max. Dosis (sertraline, fluoxetine, escitalopram) also clomipramine 2 psychotherapies (3 years, and over 6 months). 4 inpatient treatments.
4	22	Male	Single, unemployed	16	None	Intrusive thoughts, repeated checking and adjustment of body movements. YBOCS at presentation: 32 (17,15)	Many SSRIs trials, clomipramine 3x psychotherapies 1 inpatient treatment
5	63	Male	Single, disability pension	28	None	Intrusive thoughts about his existence. Aggressive thoughts. Repeated checking and mental reassurance. YBOCS at presentation: 16 (8,8)	Many SSRIs trials, clomipramine Many psychotherapies (CBT and psychoanalysis) 3 inpatient treatments
6	33	Male	Single, disability pension	22	ADHD	Intrusive thoughts, catastrophic fears about getting hurt, or doing something wrong, reacting with repeated checking and controlling. YBOCS at presentation: 33 (17,16)	SSRIs (paroxetin and sertraline), clomipramine, bupropion. Methylphenidate for ADHD. 3 psychotherapies Many inpatient treatments including treatment in multiprofessional OCD clinic.
7	40	Female	Married, unemployed	24	Anxiety, depression	Aggressive intrusive thoughts about animals, sex, children. Repeated rituals. YBOCS at presentation: 23 (20,3)	4 SSRIs trials, clomipramine, amitriptillin, augmentation with benzodiazepines, pregabaline, quetiapine 2 psychotherapies: both CBT
8	60	Male	single, disability pension	15	Depression	Intrusive thoughts about making mistakes, contamination followed by rituals of checking. YBOCS at presentation: 34 (17,17)	SSRIs (escitalopram and sertraline), clompiramine, augmentation with antipsychotics CBT for many years 6 inpatient treatments
9	43	Female	Single, unemployed	Childhood	Depression	Intrusive thoughts about cleanliness. Repeated compulsions/rituals of cleaning, ordering and controlling. YBOCS at presentation: 26 (8,18)	SSRIs (citalopram, paroxetine, fluoxetine, sertraline, and escitaopram in combination with bupropion) clomipramine 2 long-term psychotherapies (both CBT) 3 inpatient treatments
10	20	Female	Single, unemployed	10	Depression	Intrusive thoughts/fears of harm to her beloved ones. Repeated controlling and reassurance YBOCS at presentation: 33 (18,15)	SSRIs (ecitalopram, sertraline, fluoxetine, paroxetine), SNRI (venlafaxine), clomipramine, augementation with risperidone, aripiprazole. Benzodiazepines. quitiapine >2 psychotherapies with CBT. and psychoanalysis. 5 inpatient treatments.

YBOCS, Yale–Brown Obsessive Compulsive Scale; SSRI, Selective serotonin reuptake inhibitor; SNRI, Serotonin and norepinephrine reuptake inhibitors; CBT, Cognitive behavioral therapy.

Since the sample size is rather small, the results are presented mainly descriptively. According to accepted standards, the treatment response was defined as a reduction in YBOCS of at least 35% compared to baseline (55). Out of our 10 patients, 6 (2 males, 4 females) have reached the response criterion indicated by a YBOCS reduction between 42 and 100 percent at last follow-up visit (after 12 months for four patients, 9 months for one patient, and 6 months for one patient). One further patient experienced a subjectively dramatic effect on her OCD symptoms after DBS, which could also be objectified with a YBOCS-reduction of 90%. Like other patients, the optimization of the stimulation parameters for this patient took place in our clinic in an inpatient setting, and the patient was dismissed after reaching satisfactory symptom improvement. Some weeks afterwards, the patient reported deterioration of depressive symptoms and also complained about vomiting (not self-induced). The medication (clomipramine) was reduced. The patient expressed the wish to stop the stimulation or extract the electrodes. Although no clear relationship between clinical worsening and the stimulation could be established, we opted to stop the stimulation temporarily. After 3 months, the patient contacted us asking for a reactivation of the DBS-therapy.

The other 3 patients showed a slight improvement of YBOCS scores (between 8.8 and 21.9% YBOCS reduction) but did not reach the response criterion (see Table 2).

**Table 2:** Response as measured by clinical scales.

Patient number (total visits)	YBOCS Baseline	YBOCS LFU	Reduction %	Mean YBOCS baseline and before stimulation	Mean YBOCS all FU visits	Reduction %	GAF Baseline	GAF LFU	CGI-S Baseline	CGI-S LFU	CGI-I Before Stimulation	CGI-I LFU	WHOQOL Baseline	WHOQOL LFU	MADRS Baseline	MADRS LFU
1 (7)	18**	0	−100%	17	1.2	−92.9%	35	70	6	3	4	1	34	73	*	*
2 (7)	37	14	−62.2%	36	16.8	−53.3%	20	60	7	4	4	2	37	51	37	14
3 (7)	31	11	−64.5%	32	14.2	−55.6%	40	65	6	4	4	1	44	60	33	14
4 (7)	32	25	−21.9%	30	24	−20%	35	50	6	5	4	3	43	51	*	*
5 (7)	16	0	−100%	16	3.2	−80%	50	90	5	2	4	1	62	69	*	*
6 (7)	33	28	−15.1%	33	27.6	−16.4%	40	55	6	5	4	3	48	53	12	16
7 (6)	23	6	−73.9%	24.5	6.7	−72.6%	30	70	6	3	4	1	48	60	24	7
8 (6)	26	15	−42.3%	33	26.5	−19.7%	30	45	7	6	4	4	41	40	30	24
9 (5)	34	31	−8.8%	27.5	11.6	−57.8%	35	65	6	4	4	2	47	64	29	7
10 (3)	33	3	−90.9%	33	3	−90.9%	30	45	6	5	4	2	44	64	31	5
Mean	28.3	13.3	−53.0%	28.2	13.48	−52.2%	34.5	61.5	6.1	4.1	4	2	44.8	58.5	28	12.4

*missing data.

**only obsessive thoughts.

LFU, last follow-up; YBOCS, Yale–Brown Obsessive Compulsive Scale; GAF, global assessment of functioning; CGI-S, clinical global impression – severity scale; CGI-I, clinical global impression – improvement scale; WHOQOL, WHO-quality of life (BREF); scores presented here are the sumscores of all four domains. MADRS, The Montgomery–Åsberg Depression Rating Scale.

The overall mean YBOCS decreased from **28.3** at baseline to **13.3** (53% reduction) at the last follow-up. For responders (6 patients) the overall mean YBOCS decreased from **25.1** at baseline to **7.6** (69.7% reduction) at last follow-up.

**Figure 1:**
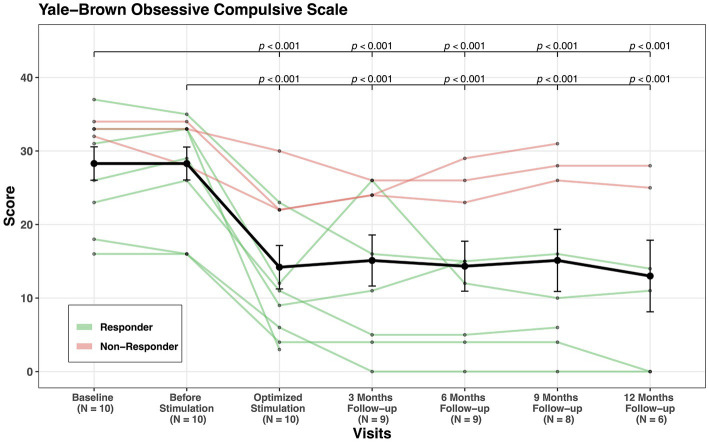
YBOCS scores’ change through visits for all patients.

Since the follow-up period varied among patients, we calculated also the mean values for YBOCS of both first two visits (baseline and before stimulation) and compared them with the mean values of all follow-up visits combined (3 months, 6 months, 9 months, and 12 months). The percentage of YBOCS reduction varied slightly as compared to the comparison between baseline and last follow-up, but the response status did not change (see Table 2; Figure 1).

**Figure 2:**
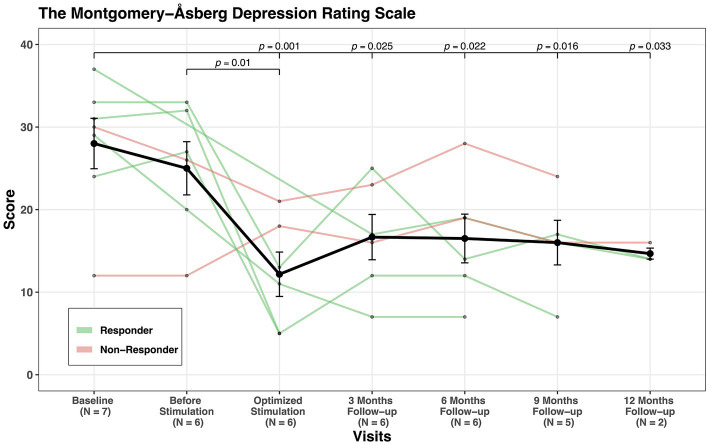
MADRS scores’ change through visits for all patients.

The improvement of the OCD symptoms was also accompanied by an improvement of depressive symptoms, as shown by a reduction in MADRS scores in six out of seven patients (MADRS scores were only available for 7 patients, see Figure 2). Overall, the mean MADRS-score improved from 28 points at baseline to 12.4 at the last follow-up.

**Figure 3:**
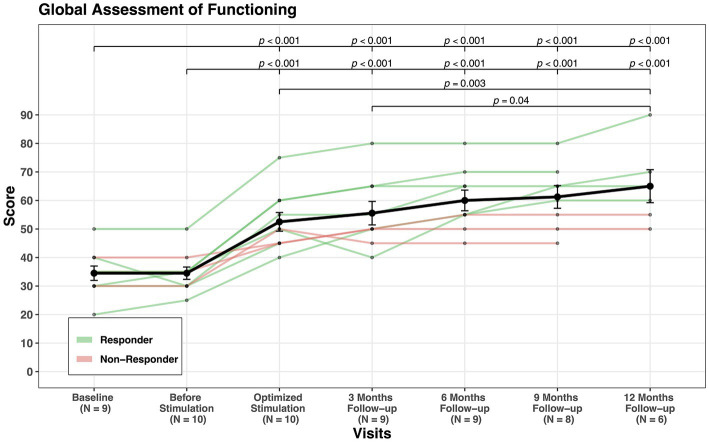
GAF scores’ change through visits for all patients.

**Figure 4:**
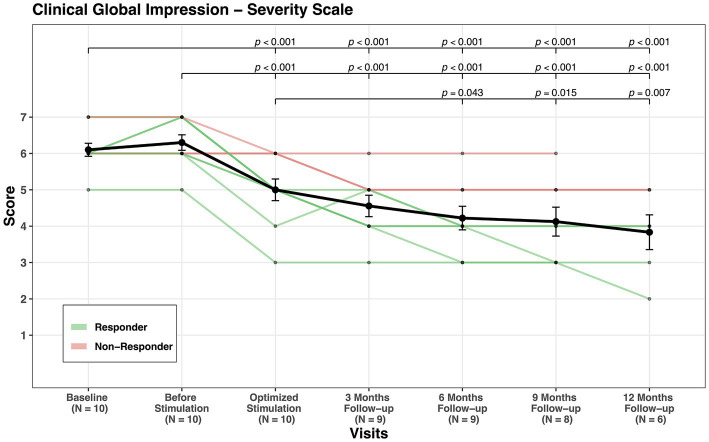
CGI-S scores’ change through visits for all patients.

**Figure 5:**
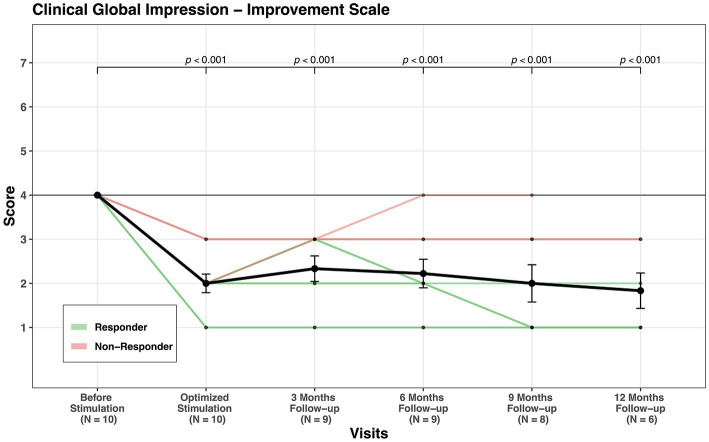
CGI-I scores’ change through visits for all patients.

GAF mean score increased from 34.5 at baseline to 61.5 at last follow-up, indicating an improvement of 78% (see Figure 3) and CGI-S showed an improvement of 32% (see Figure 4). CGI-I mean score was reduced from four points before stimulation to two points at last follow-up (see Figure 5). All domains of WHOQOL (physical, psychological, social, and environment) have shown improvement when comparing the mean values of baseline, 3 months follow-up, and last follow-up. The mean of total four domains’ scores increased from 44.8 at baseline to 54.7 at 3 months follow-up and to 58.5 at the last follow-up (see Table 3; Supplementary material).

**Table 3:** Response characteristics and side effects reported.

Patient number	Device	Year of DBS surgery	Response (Y/N)	End stimulation parameter	Medication before DBS	Medication LFU	Side effects	Occupational situation
1	IPG: PerceptPC Electrode:3391	2020	Y	6 V, 130 Hz, 60 ms	Venlafaxine 300 mg, amitriptyline 150mg, olanzapine 20 mg, haloperidol 5 mg biperiden 2 mg	Venlafaxine 150 mg and quetiapine 50 mg.	*Restlessness, sensation of fear/panic*	Started working after DBS
2	IPG: PerceptPC Electrode:3387	2021	Y	5,8 V, 160 Hz, 60 ms	fluovoxamine 100 mg, bupropion 300 mg, perazine 200 mg, amisulpride 150 mg, amfetamine 32 mg, diazepam 15 mg, risperidone 1 mg	Escitalopram 15 mg, Clomipramine 112,5 mg, bupropion 300 mg, perazine 75 mg, amisulpride 150 mg, dexamfetamine 20 mg, pregabaline 200 mg, Tilray (cannabidiole) 8 mg.	*None reported*	
3	IPG: ActivaRC Electrode:3387	2021	Y	4,6 V, 160 Hz, 120 ms	fluoxetine 60 mg	Fluoxetine 60 mg	*Insomnia, palpitaions, hypomania*	Started working after DBS
4	IPG: ActivaRC Electrode:3387	2021	N	5,2 V 130 Hz, 120 ms	paroxetin 20 mg, quetiapine 150 mg	None	Restlessness	
5	IPG: PerceptPC Electrode:3387	2021	Y	5,7 V, 130 Hz, 60 ms	escitaopram 20 mg, aripiprazole 10 mg	Escitaopram 20 mg, aripiprazole 10 mg	*Sensation of fear/panic, drowsinenss*	
6	IPG: ActivaRC Electrode:3387	2021	N	5,5 V, 130 Hz, 120 ms	paroxetine 60 mg	None	*Palpitations, impulsiveness, elevated mood, Insomnia*	
7	IPG: ActivaRC Electrode: B3301533	2022	Y	4 V, 170 Hz, 80 ms	Sertraline 50 mg, risperidone 1mg, quetiapine 50 mg, diazepam and zopiclone on demand	Sertraline 100 mg, quetiapine 50, pregabaline 200 mg	Sensation of fear/panic	Started working after DBS
8	IPG: ActivaRC Electrode: B3301533	2022	Y	4,5 V, 130 Hz, 60 ms	clomipramine 225 mg	Fluoxetine 20 mg	*Agitation, hypomania, impulsivity*	Started working after DBS
9	IPG: ActivaRC Electrode: B3301533	2022	N	5 V, 180 Hz, 60 ms	Mirtazapine 30 mg, quetiapine 225 mg, gabapentin 1,200 mg	Mirtazapine 30 mg, quetiapine 150 mg, gabapentin 1,200 mg	*Restlessness, sensation of fear/panic, Insomnia, stuttering*	
10	IPG: ActivaRC Electrode: B3301533	2022	Yes but then stimulation-stop	4,6–5,3 V, 150 Hz, 80 ms	Clomipramine 150 mg, aripiprazole 5 mg	Clomipramine 75 mg	*Sensation of fear/panic, Insomnia, nightmares*	

LFU, last follow-up.

Statistically, a significant effect of study visit was observed for each assessment inventory except for the domain environment of the WHOQOL-BREF. Post hoc tests revealed symptom improvement from baseline/before stimulation to post stimulation (see Figure 1).

The stimulation parameters were optimized individually for every patient according to symptom reduction and tolerance. The DBS parameter settings in our patients ranged from 4 to 6 V amplitude, 60–120 ms pulse width, 130–180 Hz frequency (see Table 3). Stimulation related side effects were mainly experienced during the titration visits, i.e. stimulation-induced and could be resolved by adjusting the stimulation parameters. There were no serious AEs. During the titration visits, two patients have experienced hypomanic symptoms like excessive talking, increased energy, euphoric mood, and partially inadequate behavior. Since those patients were inpatients in our clinic, the symptoms have been identified fast and the stimulation was reduced or stopped after some hours, which resulted in immediate disappearance of the hypomanic symptoms without further problems. Most frequent side effects during the optimization period were insomnia, restlessness and sensation of fear. One patient reported stuttering after about 6 months of the stimulation. The side effects are summarized in Table 3.

Three of the six responders reduced their medication after DBS. Also one of the non-responders discontinued his medication and reported no worsening of symptoms.

## 4. Discussion

The aim of this report is the evaluation of the effectiveness and safety of DBS for therapy-resistant OCD patients in a naturalistic setting. Overall, 60% of the treated patients fulfilled the response criterion, and clinical improvement continued over the follow-up period, ranging from 6 months to 1 year. In the whole sample, there was a mean reduction of 53% in the YBOCS score. The response in the YBOCS was accompanied by improvement in GAF, CGI, and quality of life.

Our results are in keeping with other published data: Denys et al. implanted 16 patients and found a YBOCS reduction of 46%, with 9 of the patients being responders (26). Barcia et al. reported response in six out of seven patients, with a median symptomatic reduction of 50% (25). Menchon et al. reported a Y-BOCS reduction of 42% with a responder rate of 60% (19). Further studies revealed comparable results, for example a ≥ 35% YBOCS reduction in four out of 6 patients for Goodman et al. (22) and a YBOCS reduction of 48% with responder rate of 70% for Luyten et al. (21). Greenberg et al. published the results of 26 patients implanted in (VC/VS), revealing clinically significant symptom reductions and functional improvement in about two-thirds of patients (23). Tyagi et al. compared VC/VS and amSTN DBS in their study, showing that stimulation at both targets was associated with a significant improvement of YBOCS scores over baseline (24).

Remarkably, the above-mentioned results lay all in the same range, even if they come from relatively small samples, and studies using various experimental designs and brain targets.

According to our results, the symptom reduction as indicated by YBOCS was paralleled by an improvement in quality of life and the regaining of social participation. In our group, four patients started to work following DBS after many years of unemployment due to illness. This illustrates the impact of DBS on patients’ level of functioning as well as overall satisfaction. This is also consistent with previous findings, reporting a significant improvement in quality of life for DBS OCD patients (39, 56).

In our group, there was one patient who showed a significant initial response and afterwards reported a deterioration of depressive symptoms and opted to stop the stimulation. Our hypothesis for this deterioration is as follows; firstly, it is well recognized that after symptom reduction or remission, many chronically ill individuals have difficulty adjusting to their new situation (57). The relief of symptoms following DBS represents a major challenge for patients, as it goes along with major changes in identity and relationships (58). Especially the regaining of social participation is accompanied by new types of challenges, such as stress at the workplace. Secondly, this patient displayed signs of a comorbid personality problem, which manifested as instability and impulsivity. Although this was not confirmed by neuropsychological assessment, it could have contributed to the afterward deterioration.

Although three patients displayed only a minor reduction of the YBOCS score and have been considered non-responders, none of them considered stopping the stimulation. One of the non-responders was able to stop his medication without deterioration of his symptoms.

Stimulation related adverse events in our group of patients could all be resolved through adjustment of the stimulation parameters. Hypomania was the most severe adverse event.

In our group of patients, there were three patients who might be considered only moderately affected according to the baseline YBOCS score (YBOCS < 24points), yet those three patients were showing clear signs of suffering, treatment resistance and evident disturbed level of functioning. They all responded well to DBS. This raises many questions or challenges to DBS research. Should DBS be restricted to only severely affected patients? What are the criteria for this severity? And how to determine the most suitable candidates for DBS surgery, in other words, which criteria determine the likelihood of a treatment response. The good response in patients with lower YBOCS score parallels to a certain extent the situation in Parkinson’s disease where the indication for deep brain stimulation moved over the years from a treatment for severely treatment resistant patients to patients in earlier stages of the disease (59).

Interestingly, in all our patients obsessive thoughts were clearly more pronounced than compulsive acts. This relativizes the relevance of the YBOCS total score as the sole criterion for the severity of the disease, as patients, who score low in compulsive acts, have a relatively low total score, even if they are extremely impaired by obsessive thoughts. The preponderance of obsessive thoughts in our patients may reflect the fact, that compulsive acts can be better addressed by CBT than obsessive thoughts. Thus, patients suffering predominantly from obsessive thoughts may be overrepresented among treatment resistant patients.

A major clinical challenge is the individual optimization of stimulation parameters. The combination of all possible settings results in a huge parameter space, making systematic testing of all combinations impossible. A valid assessment of the efficacy and tolerability of a given stimulation setting typically requires a time period of at least several days. In clinical practice, we used a stepwise exploration of contact, voltage, frequency and stimulus width. Settings that seemed optimal in the clinic were then evaluated under real life conditions, as OCD symptoms are typically context dependent.

An ongoing matter of debate is the optimal neuroanatomical target, since several neuroanatomical regions have been targeted with comparable outcomes. All our patients were implanted in the BNST. The BNST, which is considered as a part of the “extended amygdala” (60), is a brain nucleus embedding the stria terminalis and located posterior to the nucleus accumbens (61). It is suggested that the BNST is involved in striatal circuitry that integrates descending glutamatergic input with ascending modulatory inputs (62). Through its role connecting limbic forebrain structures to hypothalamic and brainstem regions associated with autonomic and neuroendocrine functions, the BNST serves as a major component in the integration of physiological and behavioral responses (63). In addition, an interaction of neurotransmitters within the BNST has been reported, primarily via a modulation of presynaptic neurotransmitter release (64–67). The BNST was first introduced as DBS target for the treatment of OCD by Nuttin et al. (68). In their study of comparing BNST DBS with NA DBS, Islam et al. reported a better outcome for DBS in the BNST (69). Yet, according to Farrand et al., the overall effect of these two brain targets was comparable (70). Another study of 24 patients found a better result of DBS in the BNST compared with patients implanted in the anterior limb of internal capsule (21) with also reported stability of symptom reduction over time (71).

An important issue in the DBS field for psychiatric disorders, which has been recently stressed (47) is the accessibility problem. Firstly, DBS for OCD requires collaboration between psychiatric and neurosurgery departments and experienced personnel for patient selection, surgery, and therapy optimization. This is only available at a few centers.

Secondly, although DBS is an approved therapy with reported long-term cost-effectiveness (72, 73), its costs are not normally covered by health insurance companies. An application for coverage of DBS’s costs for severe OCD patients is often denied by the health insurance companies. For OCD patients, who cannot afford to pay the high costs of DBS themselves, this means depriving them of access to this therapy (74).

Thirdly, DBS seems not to be perceived by many psychiatrists as an available therapeutic option. The majority of our patients were not referred to us by their psychiatrist, but presented in our clinic on their own initiative after finding the option of deep brain stimulation on the internet.

## 5. Limitations

While our data illustrate the efficacy of DBS for patients with treatment-resistant OCD, we are aware of many study limitations. First, neither randomization nor a sham control existed. Second, several patients missed some appointments, and the duration of the follow-up period varied among individuals. Thirdly, stimulation parameters were not standardized but adjusted individually for each patient. Notwithstanding these limitations, we emphasized a transparent and comprehensive presentation of the individual demographic and clinical characteristics of our patients to supply further valuable data to the DBS research field.

## 6. Conclusion

Our results further confirm that BNST DBS is effective for treatment-resistant OCD patients, as indicated by a reduction in symptoms and an overall improvement in functioning. Beside the need for additional research to define the patient’s selection criteria, the most appropriate anatomical target, and the most effective stimulation parameters, improved patient access for this therapy should be established.

## Data availability statement

The raw data supporting the conclusions of this article will be made available by the authors, without undue reservation.

## Ethics statement

The studies involving humans were approved by Ethic Committee of University of Regensburg, Germany. The studies were conducted in accordance with the local legislation and institutional requirements. The participants provided their written informed consent to participate in this study. Written informed consent was obtained from the individual(s) for the publication of any potentially identifiable images or data included in this article.

## Author contributions

MA, VL-H, TH, and BL screened the possible candidates, performed the evaluation ratings for enrolled patients. MA collected the data und summed the results. SS converted the results into figures and performed the statistical analysis. JS and DD performed the neurosurgical operations. MA, BL, and JS drafted the manuscript. Other authors revised and corrected the drafts. All the authors designed the study, interpreted the data, and approved the final version of the manuscript.

## Conflict of interest

The authors declare that the research was conducted in the absence of any commercial or financial relationships that could be construed as a potential conflict of interest.

## Publisher’s note

All claims expressed in this article are solely those of the authors and do not necessarily represent those of their affiliated organizations, or those of the publisher, the editors and the reviewers. Any product that may be evaluated in this article, or claim that may be made by its manufacturer, is not guaranteed or endorsed by the publisher.
