# Supplementary material for: Deep brain stimulation for treatment resistant obsessive compulsive disorder; an observational study with ten patients under real-life conditions
Source: Front Psychiatry. 2023 Sep 13;14:1242566. doi: 10.3389/fpsyt.2023.1242566 (PMC10533930; doi:10.3389/fpsyt.2023.1242566)
Supplement: Supplementary file 1 [file Data_Sheet_1.docx]

Supplementary Material

Figure 6 (A-D): WHOQOL-BREF domains scores’ change through visits for all patients **
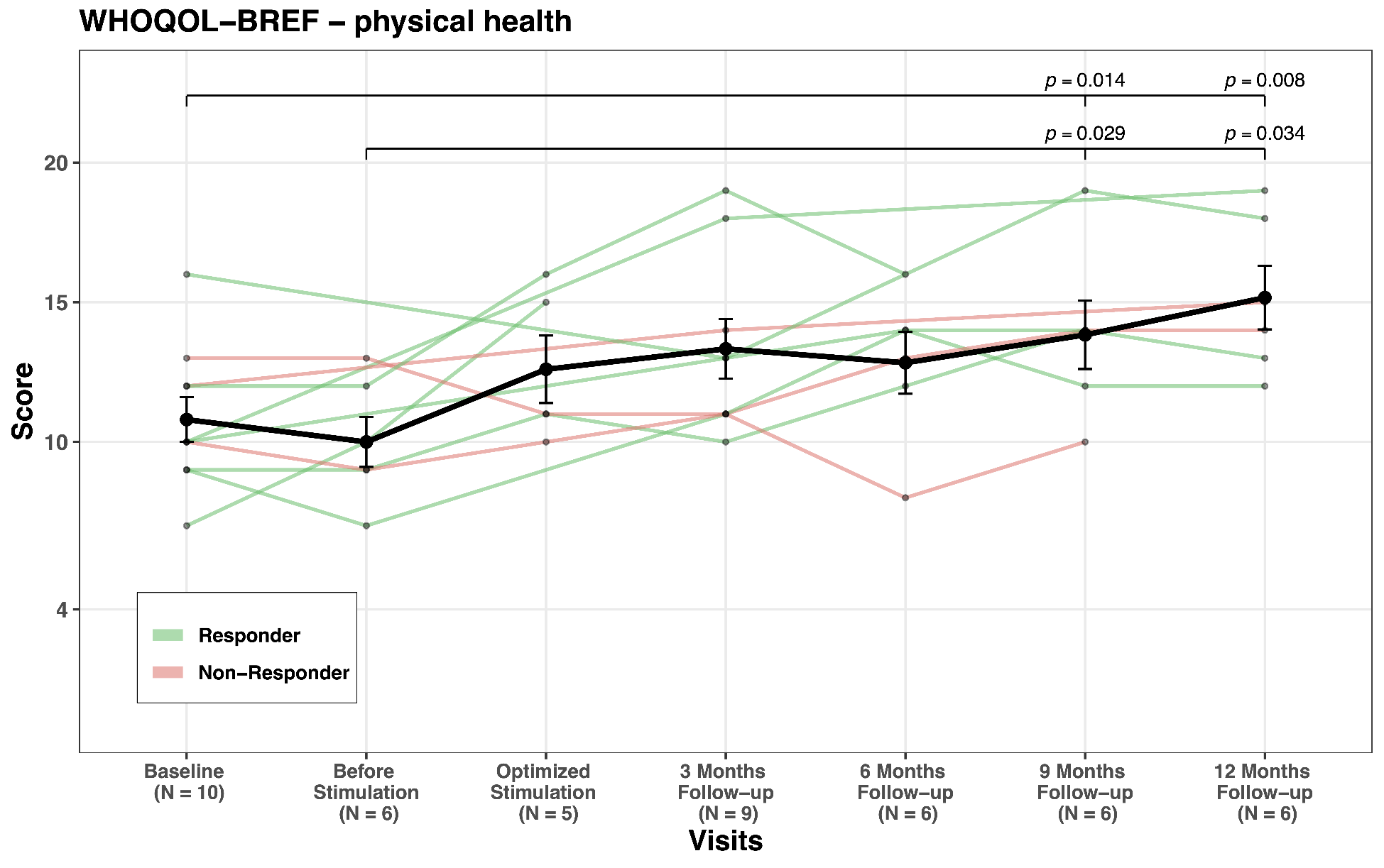


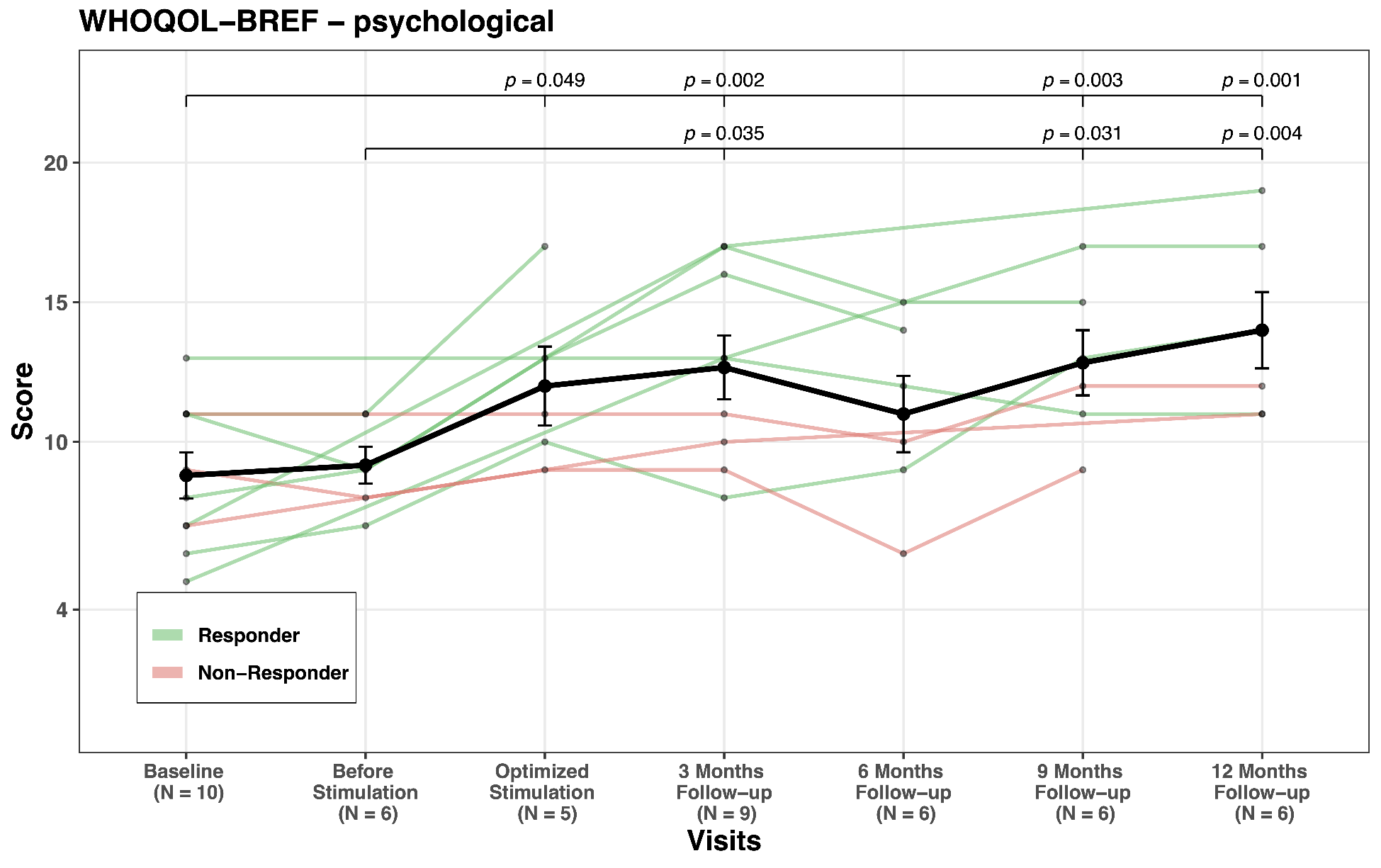

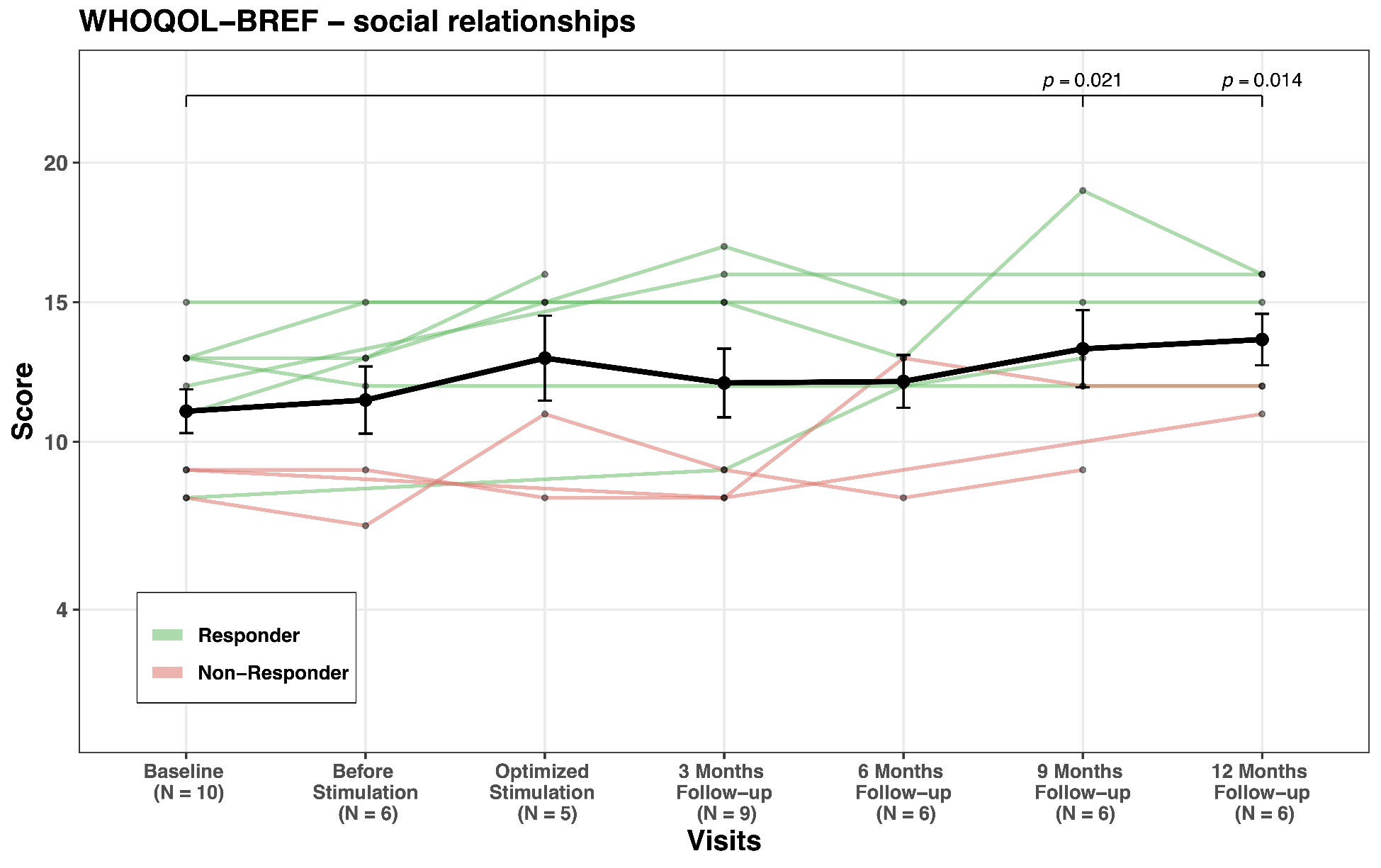

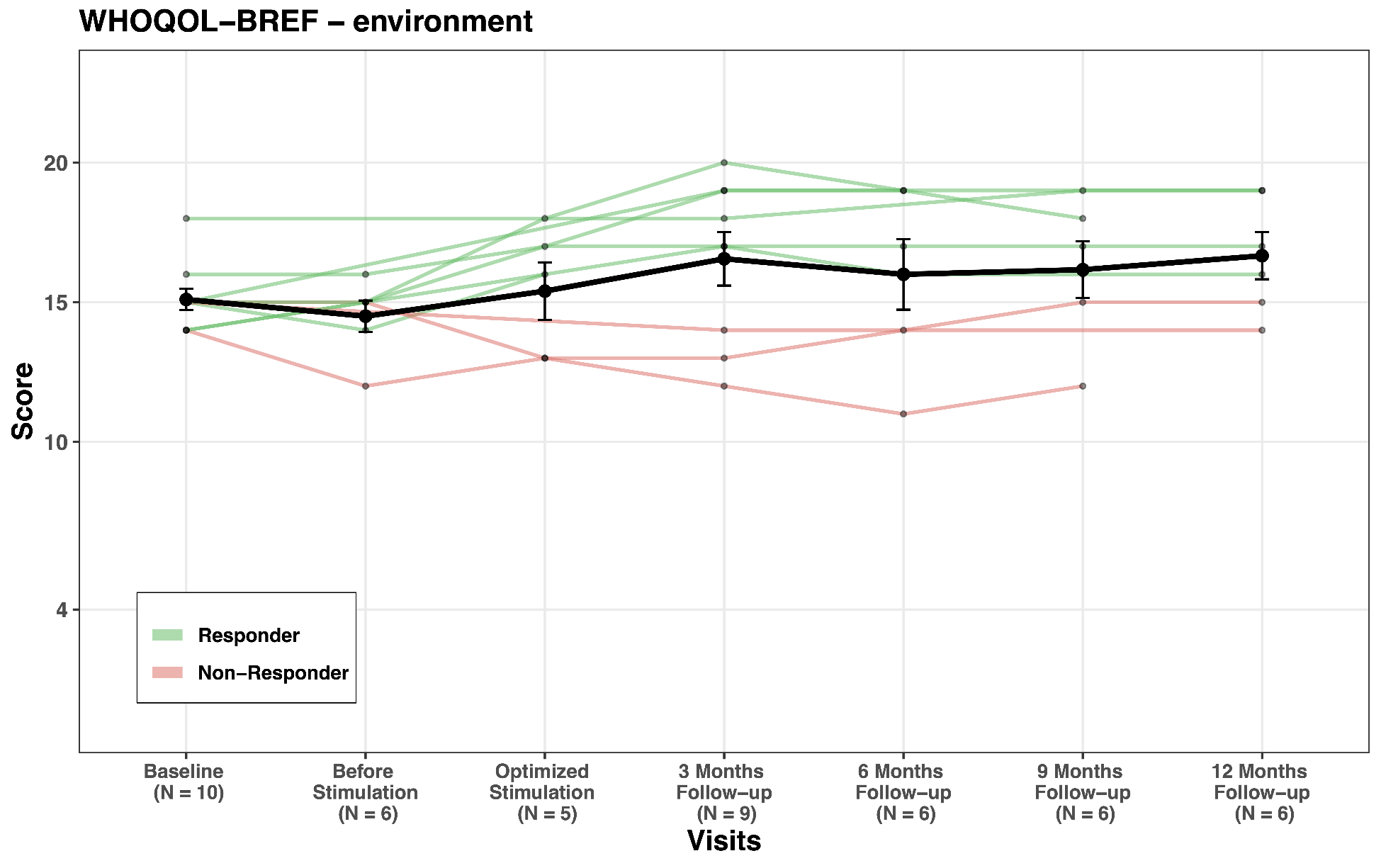


All scores of clinical assessments**

| **Patient** | **Rating** | **Baseline** | **Before stimulation** | **Optimized stimulation** | **3 months  follow-up** | **6 months  follow-up** | **9 months follow-up** | **12 months follow-up** |
| --- | --- | --- | --- | --- | --- | --- | --- | --- |
| **1** | YBOCS (O,C)  GAF CGI-S CGI-I MADRS | 18 (18,0) 35 6 -- * | 16 (16,0) 35 6 4 * | 6 (6,0) 50 4 2 * | 0 (0,0) 40 5 3 * | 0 (0,0) 55 4 2 * | 0 (0,0) 65 3 1 * | 0 (0,0) 70 3 1 * |
| **2** | YBOCS (O,C)  GAF CGI-S CGI-I MADRS | 37 (20,17) 20 7 -- 37 | 35 (18,17) 25 7 4 * | 23 (14,9) 40 5 2 * | 16 (9,7) 50 4 2 17 | 15 (8,7) 55 4 2 19 | 16 (9,7) 60 4 2 16 | 14 (7,7) 60 4 2 14 |
| **3** | YBOCS (O,C)  GAF CGI-S CGI-I MADRS | 31 (18,13) 40 6 -- 33 | 33 (19,14) 30 6 4 33 | 12 (6,6) 55 5 2 13 | 26 (11,15) 55 5 3 25 | 12 (6,6) 65 4 2 14 | 10 (5,5) 65 4 1 17 | 11 (5,6) 65 4 1 14 |
| **4** | YBOCS (O,C)  GAF CGI-S CGI-I MADRS | 32 (17,15) 35 6 -- * | 28 (15,13) 35 6 4 * | 22 (11,11) 45 6 3 * | 24 (12,12) 50 5 3 * | 23 (10,13) 50 5 3 * | 26 (13,13) 50 5 3 * | 25 (12,13) 50 5 3 * |
| **5** | YBOCS (O,C)  GAF CGI-S CGI-I MADRS | 16 (8,8) 50 5 -- * | 16 (8,8) 50 5 4 * | 4 (2,2) 75 3 1 * | 4 (2,2) 80 3 1 * | 4 (2,2) 80 3 1 * | 4 (2,2) 80 3 1 * | 0 (0,0) 90 2 1 * |
| **6** | YBOCS (O,C)  GAF CGI-S CGI-I MADRS | 33 (17,16) 40 6 -- 12 | 33 (17,16) 40 6 4 12 | 30 (15,15) 45 6 3 18 | 26 (13,13) 50 5 3 16 | 26 (13,13) 55 5 3 19 | 28 (14,14) 55 5 3 16 | 28 (14,14) 55 5 3 16 |
| **7** | YBOCS (O,C)  GAF CGI-S CGI-I MADRS | 23 (20,3) 30 6 -- 24 | 26 (16,10) 35 7 4 27 | 11 (4,7) 60 5 1 5 | 5 (0,5) 65 4 1 12 | 5 (1,4) 70 3 1 12 | 6 (2,4) 70 3 1 7 | - - - - - |
| **8** | YBOCS (O,C)  GAF CGI-S CGI-I MADRS | 34 (17,17) 30 7 -- 30 | 34 (16,18) 30 7 4 26 | 22 (11,11) 50 6 2 21 | 24 (13,11) 45 6 3 23 | 29 (16,13) 45 6 4 28 | 31 (17,14) 45 6 4 24 | - - - - - |
| **9** | YBOCS (O,C)  GAF CGI-S CGI-I MADRS | 26 (8,18) 35 6 -- 29 | 29 (12,17) 35 6 4 20 | 9 (0,9) 60 5 2 11 | 11 (3,8) 65 4 2 7 | 15 (7,8) 65 4 2 5 | - - - - - | - - - - - |
| **10** | YBOCS (O,C)  GAF CGI-S CGI-I MADRS | 33 (18,15) 30 6 -- 31 | 33 (18,15) 30 7 4 32 | 3 (3,0) 45 5 2 5 | - - - - - | - - - - - | - - - - - | - - - - - |

YBOCS: Yale–Brown Obsessive Compulsive Scale. O: obsessions’ scale. C: compulsions’ scale. GAF: global assessment of functioning. CGI-S: clinical global impression – severity scale. CGI-I: clinical global impression – improvement scale.*: missing data. -: not yet due.


 **WHOQOL-BREF**

| **Patient** |  | **Baseline** | **Before stimulation** | **Optimized stimulation** | **3 months  follow-up** | **6 months  follow-up** | **9 months follow-up** | **12 months follow-up** |
| --- | --- | --- | --- | --- | --- | --- | --- | --- |
| 1 | **D1 D2 D3 D4 Total** | 10 7 12 15 34 | * * * * * | * * * * * | 18 17 16 19 70 | * * * * * | * * * * * | 19 19 16 19 73 |
| 2 | **D1 D2 D3 D4 Total** | 10 5 8 14 37 | * * * * * | * * * * * | 14 13 9 17 53 | 13 12 12 16 53 | 12 11 12 16 51 | 12 11 12 16 51 |
| 3 | **D1 D2 D3 D4 Total** | 9 6 13 16 44 | 9 7 13 16 45 | 11 10 15 17 53 | 10 8 15 17 50 | 12 9  13 17 51 | 14 13 19 17 63 | 13  14 16 17 60 |
| 4 | **D1 D2 D3 D4 Total** | 12 7 9 15 43 | * * * * * | * * * * * | 14 10 8 14 46 | * * * * * | * * * * * | 15 11 11 14 51 |
| 5 | **D1 D2 D3 D4 Total** | 16 13 15 18 62 | * * * * * | * * * * * | 13 13 15 18 59 | * * * * * | 19 17 15 19 70 | 18 17 15 19 69 |
| 6 | **D1 D2 D3 D4 Total** | 13 11 9 15 48 | 13 11 9 15 48 | 11 11 8 13 43 | 11 11 8 13 43 | 13 10 13 14 50 | 14 12 12 15 53 | 14 12 12 15 53 |
| 7 | **D1 D2 D3 D4 Total** | 9 11 13 15 48 | 7 9 12 15 43 | * * * * * | 11 17 12 19 59 | 14 15 12 19 60 | 14 15 13 18 60 | - - - - - |
| 8 | **D1 D2 D3 D4 Total** | 10 9 8 14 41 | 9 8 7 12 36 | 10 9 11 13 43 | 11 9 9 12 41 | 8 6 8 11 33 | 10 9 9 12 40 | - - - - - |
| 9 | **D1 D2 D3 D4 Total** | 12 8 13 14 47 | 12 9 15 15 51 | 16 13 15 18 62 | 19 16 17 20 72 | 16 14 15 19 64 | - - - - - | - - - - - |
| 10 | **D1 D2 D3 D4 Total** | 7 11 11 15 44 | 10 11 13 14 48 | 15 17 16 16 64 | - - - - - |  |  |  |

WHOQOL: WHO-quality of life. D1: Domain „physical health“. D2: Domain „psychological“. D3: Domain „social“. D4: Domain „environmental“.*: missing data. -: not yet due.
